# Individual and community level determinants of delayed antenatal care initiation in Ethiopia: A multilevel analysis of the 2019 Ethiopian Mini Demographic Health Survey

**DOI:** 10.1371/journal.pone.0300750

**Published:** 2024-05-16

**Authors:** Addisu Alehegn Alemu, Liknaw Bewket Zeleke, Desalegn Abebaw Jember, Getachew Mullu Kassa, Marjan Khajehei

**Affiliations:** 1 School of Women’s and Children’s Health, University of New South Wales Sydney, Kensington, Australia; 2 College of Health Sciences, Debre Markos University, Debre Markos, Ethiopia; 3 St. Paul Hospital Millennium Medical College, Addis Ababa, Ethiopia; 4 Women’s and Newborn Health, Westmead Hospital, Westmead, Australia; 5 The University of Sydney, Sydney, Australia; 6 Western Sydney University, Sydney, Australia; Public Library of Science, UNITED KINGDOM

## Abstract

**Background:**

Antenatal care (ANC) is essential health care and medical support provided to pregnant women, with the aim of promoting optimal health for both the mother and the developing baby. Pregnant women should initiate ANC within the first trimester of pregnancy to access a wide range of crucial services. Early initiation of ANC significantly reduces adverse pregnancy outcomes, yet many women in Sub-Saharan Africa delay its initiation. The aim of this study was to assess prevalence and determinants of delayed ANC initiation in Ethiopia.

**Methods:**

We conducted a secondary data analysis of the 2019 Ethiopian Mini Demographic and Health Survey (EMDHS). The study involved women of reproductive age who had given birth within the five years prior to the survey and had attended ANC for their most recent child. A total weighted sample of 2,895 pregnant women were included in the analysis. Due to the hierarchical nature of the data, we employed a multi-level logistic regression model to examine both individual and community level factors associated with delayed ANC initiation. The findings of the regressions were presented with odds ratios (OR), 95% confidence intervals (CI), and p-values. All the statistical analysis were performed using STATA—14 software.

**Results:**

This study showed that 62.3% (95% CI: 60.5, 64.1) of pregnant women in Ethiopia delayed ANC initiation. Participants, on average, began their ANC at 4 months gestational age. Women with no education (AOR = 2.1; 95% CI: 1.4, 3.0), poorest wealth status (AOR = 1.9; 95% CI: 1.3, 2.8), from the Southern Nations, Nationalities, and Peoples (SNNP) region (AOR = 2.1; 95% CI: 1.3, 3.3), and those who gave birth at home (AOR = 1.4; 95% CI: 1.1, 1.7) were more likely to delay ANC initiation.

**Conclusions:**

The prevalence of delayed ANC initiation in Ethiopia was high. Enhancing mothers’ education, empowering them through economic initiatives, improving their health-seeking behavior towards facility delivery, and universally reinforcing standardized ANC, along with collaborating with the existing local community structure to disseminate health information, are recommended measures to reduce delayed ANC initiation.

## Introduction

Pregnancy and childbirth are pivotal moments, but they bring heightened vulnerability for both mothers and their unborn children [1]. Every day, over 800 maternal deaths occur globally, with 99% of these occur in low and middle income countries [2, 3]. However, different interventions and strategies prevent disability and deaths during pregnancy and childbirth [4]. Antenatal Care (ANC) constitutes one of the fundamental elements of the safe motherhood initiatives [5]. ANC is a crucial opportunity and key strategy to reduce maternal and child mortality, which is prevalent in low and middle-income countries [6–8].

World Health Organization (WHO) describes ANC as the healthcare provided to pregnant women to improve the well-being of both mothers and their unborn children [3]. It encompasses a range of preventive and therapeutic healthcare services provided throughout the course of a pregnancy. The service includes preventing complications, early detection and management of underlying conditions, diagnosing and addressing sexually transmitted infections, and preventing the fetus from Human Immunodeficiency Virus (HIV) and Hepatitis B infections [9, 10]. It also increases the likelihood of skilled birth attendance and utilization of postnatal care [11]. However, WHO suggests a minimum of eight ANC sessions, with the first ideally starting in the first trimester of pregnancy to maximize its benefits [10]. The timing of the first ANC visit has been shown to influence the provision of all the recommended components of the service [12, 13]. Studies show that early first ANC attendance results in receiving the recommended services, whereas delayed visits lead to incomplete services [14]. Similarly, it has been evidenced that pregnant women who start ANC early are at a lower risk of experiencing adverse obstetric outcomes compared to those who delay the initiation of ANC [15, 16].

Despite the advantages of early ANC initiation, delayed initiation of ANC in sub-Saharan Africa, including Ethiopia, remains common [17, 18]. In Ethiopia, significant improvement has been achieved in ensuring that at least one ANC visit is attended [19, 20], but a considerable number of women delay their initial ANC visit [21]. According to the study analyzing the 2016 Ethiopian Demographic and Health Survey, 67.31% of ANC users initiated their first ANC visit after 12 weeks of pregnancy [22]. Delayed ANC is common in Ethiopia as a result of various factors, including demographics, educational disparities, cultural influences, economic constraints, and geographical obstacles [21, 22]. Ethiopia has made significant progress in reducing maternal mortality, decreasing the rate from 871 to 412 deaths per 100,000 over a span of sixteen years (2000–2016) [20]. The success is credited to improved health system development involving intersectoral collaboration, community-centered healthcare, data-informed policy-making, strong cooperation with local and national authorities, and steadfast high-level political commitment [20, 23, 24]. Despite this significant achievement, Ethiopia still contends with one of the world’s highest maternal mortality rates [2], and only 35.4% of women receive their initial ANC check-up within the first trimester of pregnancy [25]. In this sense, a considerable number of pregnant women in Ethiopia may experience adverse effects due to the delayed commencement of essential services. Therefore, this study was conducted to assess the prevalence and determinants of delayed initiation of antenatal care in Ethiopia using nationally representative data.

## Methods

### Data source

**Fig 1:**
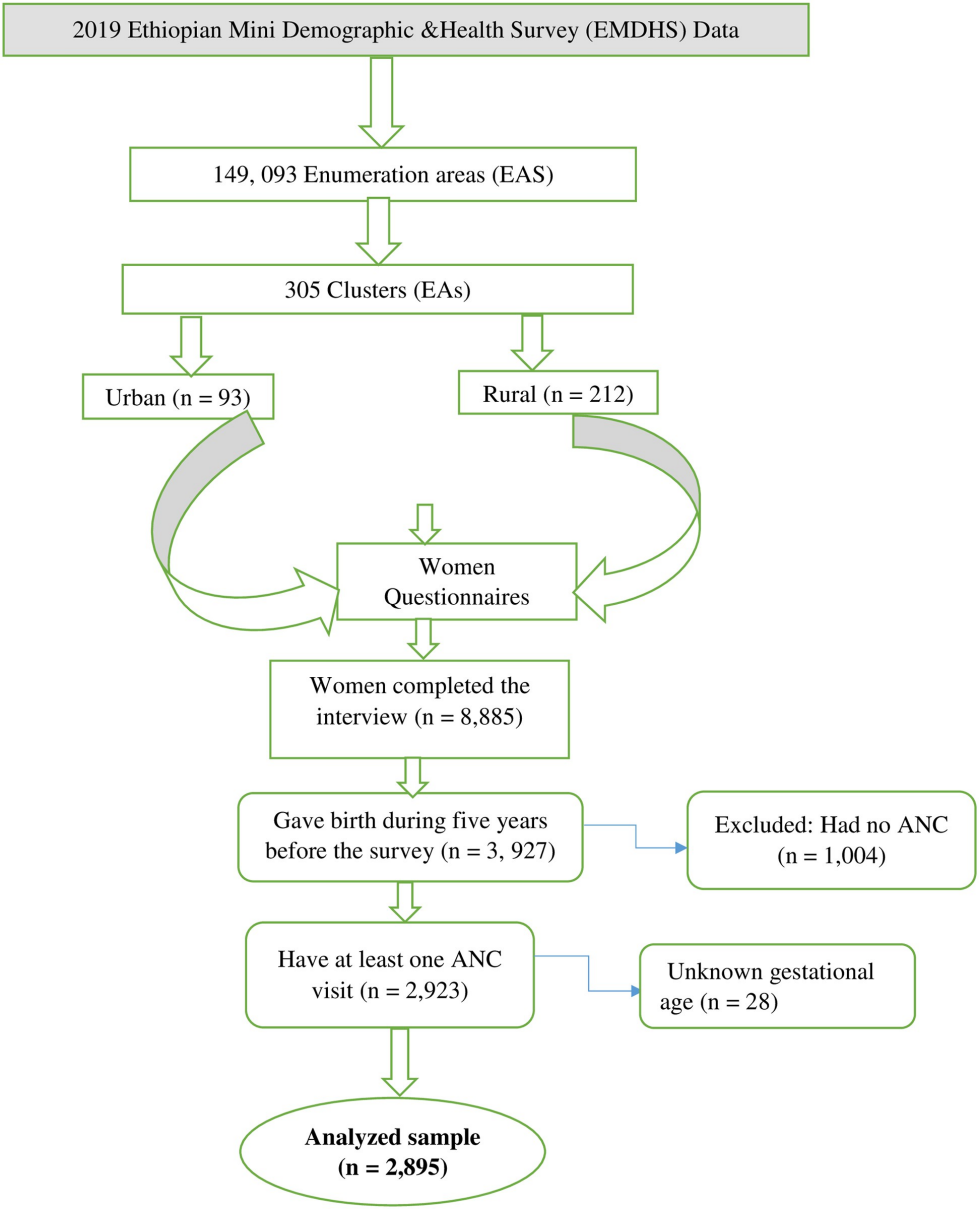
Schematic presentation of the selection of participants from the 2019 EMDHS data.

In this study, DHS data were employed, which is a nationally representative cross-sectional survey. DHS measures essential indicators that are crucial for countries to collect data to inform their policies and practices. It maintains consistency in data collection procedures, sampling methods, questionnaires, and coding, ensuring that the results can be compared across different countries. The study participants were chosen using a two-stage stratified sampling technique as per the DHS approach. The data was sourced from the DHS website (www.dhsprogram.com) after obtaining a formal permission. The 2019 EMDHS, which was a survey conducted among 8,663 households, ensuring representation of the country. The survey involved interviews with 8,885 women aged 15 to 49 years and had a response rate of 98.6%. Our analysis was confined to a group of 3,927 women who had given birth within the five years prior to the survey. Within this group, those who did not attend any ANC visits (n = 1,004) were excluded from the analysis. The remaining 2,923 women were assessed for the timing of their first ANC visit during their most recent pregnancy, and those who could not provide their gestational age (n = 28) were also excluded. In the end, the study encompassed a total of 2,895 maternal records (Fig 1). Comprehensive information regarding the survey has been previously documented elsewhere [19].

### Variables of the study

#### Outcome variable

The outcome variable of this study was delayed ANC initiation. Delayed antenatal care refers to a situation in which pregnant women initiate their ANC service after the first 12 weeks of pregnancy [10].

#### Independent variables

The independent variables were categorized into individual and community level variables. The individual level variables included women’s age, religion, parity, educational status, wealth status, marital status, family size, number of living children, age at their first childbirth, contraceptive use and intention to use, post-natal care utilization, and place of delivery.

The community-level variables of interest included place of residence and geographic region.

### Data processing and analysis

The extracted data were carefully coded and entered into the statistical database for analysis. Furthermore, the data underwent a process of adjustment through the application of sampling weight (v005), primary sampling unit (v023), and strata (v021) in order to achieve a reliable statistical estimate. Data analysis was performed using STATA-14 software (STATA Corporation, College Station, TX, USA). Descriptive statistics were employed to summarize frequency and percentage data. A multi-level logistic regression analysis technique was utilized to identify predictors of delayed ANC initiation. The association of each predictor variable with the outcome variable was assessed at 95% CI considering a 5% significance level. Both individual and community level variables were considered in the model after bivariate analysis and a p-value of < 0.05. Additionally, the presence of multicollinearity among independent variables was assessed using the Variance Inflation Factor (VIF) at a cut-off point of 10. Variables having a VIF value of less than 10 indicated the absence of multicollinearity [26]. In the final multi-level models, the association between all predictor variables and delayed ANC initiation was determined, considering a p-value of < 0.05. The results were presented using adjusted odds ratio (AOR) with a 95% CI.

Four models were conducted in this analysis. The first model (empty model) was used to estimate the random variability in the intercept. The second model assessed the impact of individual-level factors on delayed ANC initiation. The third model evaluated the effect of community-level factors on delayed ANC initiation. Finally, the fourth model estimated the effect of both individual- and community-level factors on delayed ANC initiation. The Intra-Cluster Correlation (ICC) was included to illustrate cluster correlation within each model. The Proportional Change in Variance (PCV) was also calculated to determine the contributions of variables in predicting delayed ANC initiation in each model. The model with the highest PCV value was considered to identify the factors associated with delayed ANC initiation. Variables with a p-value < 0.05 were considered as significantly associated with delayed ANC initiation. As for model fit statistics, the Akakie Information Criterion (AIC) was used to assess the goodness of fit of the adjusted final model in comparison to the preceding models (Individual and community-level model adjustments). The AIC value for each subsequent model was compared and the model with the lowest value was considered to be the best fit model [27].

### Ethics approval

The 2019 EMDHS was conducted after obtaining ethical clearance from Ethiopia Health and Nutrition Research Institute Review Board, the Ministry of Science and Technology, the Institutional Review Board of ICF International, and the Centers for Disease Control and Prevention. For this specific research, permission was given by the Demographic and Health Surveys Program to access the data after a review of the submitted brief descriptions of the study to the DHS program. The datasets were treated with the utmost confidentiality.

## Results

### Sociodemographic characteristics of participants

This study involved data from a weighted sample of 2,895 women aged between 15 and 49 years who had given birth within the five years before the survey and had attended ANC during their last pregnancy. On average, the participants in the study began their first ANC visit at approximately 4 months of their pregnancy. Majority of women (70.1%) were rural residents and less than half (41.6%) were Orthodox followers. More than two-third (37.0%) of the participants were from the Oromia region followed by those from the Amhara region (24.0%). The majority of mothers aged between 20 and 34 years (73.8), were married (93.5%) and had a family size of less than 6 (54.6%). More than two out of five mothers (43.7%) had no formal education. The socio-demographic characteristics of the study participants are presented in Table 1.

**Table 1:** Sociodemographic characteristics of the study population.

Variable	Number (n = 2,895)	Percentage
**Residence**		
Urban	866	29.9
Rural	2,029	70.1
**Religion**		
Orthodox	1,204	41.6
Protestant	785	27.1
Muslim	880	30.4
Others	26	0.9
**Region**		
Tigray	271	9.4
Afar	32	1.1
Amhara	696	24.0
Oromia	1069	37.0
Somali	66	2.3
Benshangul	39	1.3
SNNPR	557	19.2
Gambella	16	0.6
Harari	9	0.3
Addis Ababa	122	4.2
Dire Dawa	17	0.6
**Marital status**		
Single	188	6.5
Married	2,707	93.5
**Mother’s age**		
≤ 19	153	5.3
20–34	2,135	73.8
≥35	607	21.0
**Mother’s education**		
No education	1,264	43.7
Primary	1,148	39.7
Secondary	330	11.4
Higher	152	5.3
**Wealth status**		
Poorest	398	13.8
Poorer	575	19.9
Medium	585	20.2
Richer	572	19.8
Richest	765	26.4
**Family size**		
1–5	1,581	54.6
≥ 6	1,314	45.4

### Obstetrics characteristics of the participants

Almost two-thirds of the women (61.5%) gave birth for the first time at the age of 19 or younger. Nearly half (46.8%) and 65.8% of the study participants had given birth to 2–4 children and gave birth at a health facility, respectively. Less than one fifth (17.4%) of women received postnatal care service within two months of postpartum period. Majority of the participants (98.9%) were informed about at least one modern family planning method. Moreover, the prevalence of delayed ANC initiation was 62.3% (95% CI: 60.5, 64.1). The information regarding the obstetric and gynecological characteristics of the study participants is found in Table 2.

**Table 2:** Obstetric and gynecologic related characteristics of the study participants in Ethiopia.

Variables	Number (n = 2,895)	Percentage
**Woman’s age at 1st birth**		
≤ 19	1780	61.5
≤ 20	1115	38.5
**Birth order**		
One	684	23.6
2–4	1356	46.8
≥ 5	855	29.6
**Total number of living children**		
≤ 4	2,168	74.9
≥ 5	727	25.1
**Place of delivery**		
Home	991	34.2
Facility	1904	65.8
**Post-natal care service utilization**		
Yes	505	17.4
No	2,390	82.6
**Time of first ANC check-up**		
Before 12 weeks	1,092	37.7
12 weeks and above	1,803	62.3
**Awareness of any modern family planning method**		
Yes	2,862	98.9
No	33	1.1
**Contraceptive use and intention**		
Using modern method	1,434	49.2
Using traditional method	16	0.1
Does not intend to use	1,445	49.7

### Predictors of delayed antenatal care initiation

Multilevel logistic regression analysis was conducted to identify predictors of delayed ANC initiation at both individual and community levels using the 2019 EMDHS data. In Model 1 which is also referred to as the empty model, only a random intercept was included to account for variations between clusters. In this model, 21.4% of the total variance in the odds of delayed ANC initiation was explained by between-cluster variation (ICC = 21.4%, P<0.001). Similarly, the ICC was computed in each successive model to grasp the relative impacts of individual and community-level factors on delayed ANC initiation. The between-cluster variability decreased across successive models, dropping from 21.4% in the empty model to 12.0% in the individual-level model, 9.2% in the community-level model, and 7.4% in the combined model. As per the findings of the PCV, the inclusion of predictor variables in the empty model provided a better explanation for the predictors of delayed ANC initiation. The PCV result for Model II was 30%, for Model III was 39%, and for Model IV was 47%. Model IV suggested that 47% of the variations in the delayed ANC initiation between communities were explained by both the individual and community-level factors. As per the best fitted model (Model IV) maternal educational status, wealth status, place of birth, and geographical region were found to be significantly associated with delayed ANC initiation.

After adjusting for other explanatory variables, mothers with no education and primary education were 2.1 and 1.6 times more likely to delay ANC initiation compared to those with higher education (AOR: 2.1, 95% CI: 1.4, 3.0) and (AOR: 1.6, 95% CI: 1.1, 2.3), respectively. Mothers who belong to the poorest and poorer socioeconomic status were 90% more likely to delay their ANC initiation, with (AOR:1.9, 95% CI: 1.3, 2.8) for both, compared to mothers with the richest socioeconomic status. The odds of delayed ANC initiation were 40% higher for mothers who had a home birth compared to those with a facility birth (AOR: 1.4, 95% CI: 1.1, 1.7).

The likelihood of delayed initiation of ANC among women from the Afar, Harari, and Dire Dawa regions was decreased with (AOR: 0.6; 95% CI: 0.4, 0.9), (AOR: 0.4; 95% CI: 0.3, 0.7), and (AOR: 0.4; 95% CI: 0.2, 0.6) respectively, compared to those from Tigray. Conversely, women from S.N.N.P had a higher likelihood of delayed initiation, with (AOR: 2.1; 95% CI: 1.3, 3.3) compared to Tigray (Table 3).

**Table 3:** Predictors of delayed antenatal care visit initiation.

Variable	Model I	Model II (Individual level)		Model III (Community level)		Model IV (Combined level)	
		AOR (95% CI)	P-value	AOR (95% CI)	P-value	AOR (95% CI)	P-value
**Educational status**							
No education		2.0 (1.3, 2.9)	0.001			2.1 (1.4, 3.0)	<0.001
Primary		1.6 (1.2, 2.4)	0.006			1.6 (1.1, 2.3)	0.009
Secondary		1.5 (1.0, 2.2)	0.060			1.4 (0.9, 2.1)	0.078
Higher		Ref.				Ref.	
**Wealth status**							
Poorest		2.8 (2.1, 3.9)	<0.001			1.9 (1.3, 2.8)	0.001
Poorer		2.2 (1.6, 3.0)	<0.001			1.9 (1.3, 2.8)	0.114
Middle		2.2 (1.6, 3.0)	<0.001			1.4 (0.9, 2.0)	0.135
Richer		1.9 (1.4, 2.6)	<0.001			1.3 (0.9, 1.9)	0.271
Richest		Ref.				Ref.	
**Family size**							
1–5		Ref.				Ref.	
≥ 6		1.1 (0.9, 1.4)	0.326			1.1 (0.9, 1.4)	0.331
**Age at first birth**							
≤ 19		1.1(0.9, 1.3)	0.405			1.0 (0.9, 1.3)	0.503
≥20		Ref.				Ref.	
**Birth order**							
One		Ref.				Ref.	
2–4		1.1 (0.9, 1.4)	0.403			1.1 (0.9, 1.3)	0.448
≥ 5		1.1 (0.7, 1.8)	0.534			1.1 (0.7, 1.7)	0.586
**Total number of living children**							
≤ 4		Ref.				Ref.	
≥ 5		1.0 (0.7, 1.6)	0.740			1.0 (0.6, 1.5)	0.810
**Place of birth**							
Home		1.5 (1.2, 1.8)	<0.001			1.4 (1.1, 1.7)	0.001
Facility		Ref.				Ref.	
**Contraceptive use and intention to use**							
Using modern method		Ref.				Ref.	
Using traditional method		1.3 (0.5, 3.5)	0.564			1.4 (0.5, 3.7)	0.502
Does not intend to use		1.0 (0.8, 1.2)	0.782			1.1 (0.8, 1.3)	0.568
**Residence**							
Urban				Ref.		Ref.	
Rural				2.0 (1.6, 2.7)	<0.001	1.2 (0.8, 1.7)	0.347
**Region**							
Tigray				Ref.		Ref.	
Afar				0.9 (0.6, 1.53)	0.720	0.6 (0.4, 0.9)	0.024
Amhara				0.9 (0.6, 1.4)	0.554	0.8 (0.5,1.3)	0.337
Oromia				1.4 (0.9, 2.1)	0.182	1.3 (0.8, 1.9)	0.306
Somali				1.7 (0.9, 3.3)	0.121	1.0 (0.5, 2.0)	0.993
Benishangul				1.2 (0.8, 2.0)	0.416	1.1 (0.7, 1.8)	0.593
SNNP				2.2 (1.4, 3.5)	0.001	2.1 (1.3, 3.3)	0.002
Gambella				1.26 (0.8, 2.0)	0.355	1.2 (0.8, 2.0)	0.403
Harari				0.4 (0.2, 0.7)	<0.001	0.4 (0.3, 0.7)	<0.001
Addis Ababa				0.6 (0.3, 0.9)	0.044	0.6 (0.4, 1.1)	0.107
Dire Dawa				0.4 (0.3, 0.7)	0.001	0.4 (0.2, 0.6)	<0.001
**Random effects**							
**Community variance (SE)**	0.9 (0.1)	0.7 (0.1)		0.6(0.1)		0.5(0.1)	
**ICC**	**21.4%**	**12.0%**		**9.2%**		**7.4%**	
**PCV**	Ref.	30%		39%		47%	
Model Fit Statistics							
**Log-likelihood**	-1892.8968	-1819.1613		-1822.2541		-1783.5866	
**AIC**	3789.794	3672.323		3670.508		3623.173	
**BIC**	3801.744	3773.901		3748.186		3790.479	

**PCV** = Proportional Change in Variance, was calculated for successive models with reference to null model to look at relative contribution of each model to explain delayed ANC attendance.

**ICC** = Intra Cluster Correlation Coefficient, **AIC** = Akakian Information Criteria, and **SE** = Standard Error.

## Discussion

The present study has provided strong evidence regarding the prevalence and associated factors of delayed ANC initiation in Ethiopia, a nation grappling with significant maternal and newborn health challenges [19, 20]. To the best of our knowledge, this research constitutes the latest DHS dataset in the country, focusing on the nationwide investigation of delayed ANC initiation, thus offering invaluable insights for crafting interventions aimed at mitigating maternal and newborn health issues. It is also apparent that there is a notable lack of data concerning the timing of ANC initiation [28].

The current study revealed that 62.3% of mothers in Ethiopia delayed their ANC initiation. This finding is lower than that reported in other studies conducted in different parts of the country [14, 29, 30], Nigeria [31] and Zambia [32]. The observed difference between the current and the previous studies could be attributed to variation in study period, setting and sample size as speculated by other studies [33, 34]. Globally, there has been demonstrated and consistent improvement in the early initiation of ANC [28]. However, the prevalence of delayed ANC initiation in the current study is higher than other studies conducted in different parts of the country [14, 35] and Myanmar [36]. This disparity may be attributed to the fact that the previous studies were conducted in cities where the majority of participants might have better community awareness, health care access and service utilization. In contrast the current study predominantly included rural residents. Evidences showed that ANC utilization is influenced by residence and exposure to media [8, 37, 38]. These studies revealed that urban residence and media exposure were associated with an increased uptake of ANC.

In this study, mothers without education and with primary education were 2.1 and 1.6 times more likely to delay ANC, respectively, compared to mothers who attended secondary education. This finding is supported by others studies conducted in Ethiopia [21, 22], South Africa [39], Nigeria [31] and Zambia [32]. This could be because education increases awareness of the importance of timely ANC initiation and the risks associated with delayed ANC initiation [25, 40]. Education equips individuals with the ability to make informed decisions about healthcare services and enhances their financial independence for accessing medical care [19, 38].

The current study revealed that mothers who belong to the poorest and poorer socioeconomic statuses were 90% more likely to delay their ANC initiation compared to those with the richest socioeconomic status. This finding supports the results of studies conducted in Ethiopia [22], Ghana [33], and India [41, 42]. This might be due to the out-of-pocket expenditure associated with the service. Women are responsible for transportation and other indirect costs to receive the service, even though the service is offered free of charge [20, 43]. In this context, wealthier women can manage transportation and work commitments, facilitating timely ANC. In contrast, less affluent women might delay ANC due to fear of unexpected payments and prioritizing daily expenses over their health [44, 45].

Mothers who had a home birth were 40% more likely to delay ANC initiation compared to those who gave birth in a facility. This result is consistent with previous studies in the country [17, 45], Bangladesh [46], and Ghana [47]. This may be because women who choose home births often live in remote areas, have a lower socioeconomic status, and possess lower levels of education [48]. The study’s design does not explicitly indicate the cause-and-effect relationship it could also be because delayed ANC increases the probability of women opting for home births instead of facility-based deliveries [49]. Delayed ANC reduces the odds of mothers receiving comprehensive antenatal services [50], disrupting the continuity of maternity care [51] and potentially resulting in an increase in home births [52].

This study revealed that the likelihood of delayed ANC initiation decreased for women from the Afar, Harari, and Dire Dawa regions compared to those from Tigray. Conversely, women from SNNP had higher odds of delayed initiation compared to Tigray. The better readiness of health care facilities to provide standardized ANC in Dire Dawa [53], the traditional information sharing system (Dagu) in Afar [54], and the relatively higher wealth status of the population in Harari [20] could be among the possible reasons for the observed difference. Unlike women in the Tigray region, the primary source of information about ANC for women in the Afar region was Dagu [55]. Similarly, the higher odds of delayed ANC initiation in SNNP can be justified with the evidenced lower media exposure of the population compared to the Tigray [20]. Media holds the potential to effectively convey healthcare information when the quality and reliability of exchanged information are under surveillance [56].

The strengths of the study encompass utilizing the latest nationally representative dataset with appropriate weighting and employing a model that accounts for the data’s hierarchical structure. However, the study had several shortcomings and limitations. The primary limitations of this study included the risk of recall bias, as participants had to recall information about their ANC experiences in the last five years prior to the survey. Likewise, reporting timing of service initiation in months could lead to less precise estimates.

## Conclusions and recommendations

Our study revealed that delayed ANC initiation in Ethiopia was high. Delaying the initiation of ANC was linked to lower levels of maternal education, lower socio-economic status, giving birth at home, and residing in the Southern Nations, Nationalities, and Peoples region. Therefore, enhancing mothers’ education, empowering them through economic initiatives, improving their health-seeking behavior towards facility delivery, and universally reinforcing standardized ANC, along with collaborating with the existing local community structure to disseminate health information, are recommended measures to reduce the prevalence of delayed ANC initiation in the country. It is evidenced that targeting those facing economic challenges is important to improve the utilization of such preventive healthcare services [57]. Furthermore, future qualitative studies are recommended to have deeper understanding of factors influencing pregnant women’s ANC initiation decisions.

## Abbreviations

AIC: Akakie Information Criterion
ANC: Antenatal care
AOR: Adjusted odds ratio
CI: Confidence Interval
DHS: Demographic and Health Survey
EAs: Enumeration Areas
EMDHS: Ethiopian Mini Demographic and Health Survey
ICC: Intra Cluster Correlation Coefficient
PCV: Proportional Change in Variance
SE: Standard Error
SNNP: Southern Nations, Nationalities and Peoples
VIF: Variance Inflation Factor
WHO: World Health Organization

## References

[1] Okedo-Alex IN, Akamike IC, Ezeanosike OB, Uneke CJ. Determinants of antenatal care utilisation in sub-Saharan Africa: a systematic review. BMJ open. 2019;9(10):e031890. doi: 10.1136/bmjopen-2019-031890 31594900 PMC6797296

[2] WHO. Trends in maternal mortality 2000 to 2020: estimates by WHO, UNICEF, UNFPA, World Bank Group and UNDESA/Population Division. 2023.

[3] Tunçalp Ӧ, Pena-Rosas JP, Lawrie T, Bucagu M, Oladapo OT, Portela A, et al. WHO recommendations on antenatal care for a positive pregnancy experience-going beyond survival. Bjog. 2017;124(6):860–2. doi: 10.1111/1471-0528.14599 28190290

[4] World Health Organization. Maternal mortality 2020. https://www.who.int/news-room/fact-sheets/detail/maternal-mortality.

[5] World Health Organization. Ending preventable maternal mortality (EPMM) 2020. https://www.who.int/initiatives/ending-preventable-maternal-mortality#:~:text=Guiding%20principles%20for%20EPMM&text=Ensure%20country%20ownership%2C%20leadership%20and,to%20all%20who%20need%20it.

[6] Organization WH. WHO recommendations on antenatal care for a positive pregnancy experience: summary: highlights and key messages from the World Health Organization’s 2016 global recommendations for routine antenatal care. World Health Organization, 2018.

[7] Tey N-P, Lai S-l. Correlates of and barriers to the utilization of health services for delivery in South Asia and Sub-Saharan Africa. The Scientific World Journal. 2013;2013. 10.1155/2013/423403 PMC3830882 24288482

[8] Wolde HF, Tsegaye AT, Sisay MM. Late initiation of antenatal care and associated factors among pregnant women in Addis Zemen primary hospital, South Gondar, Ethiopia. Reproductive health. 2019;16:1–8. 31151402 10.1186/s12978-019-0745-2 PMC6544982

[9] Organization WH. Prevention of mother-to-child transmission of hepatitis B virus (HBV): Guidelines on antiviral prophylaxis in pregnancy: World Health Organization; 2020. 32833415

[10] Organization WH. WHO recommendations on antenatal care for a positive pregnancy experience: World Health Organization; 2016. 28079998

[11] Gitonga E, Muiruri F. Determinants of health facility delivery among women in Tharaka Nithi county, Kenya. The Pan African Medical Journal. 2016;25(Suppl 2). doi: 10.11604/pamj.supp.2016.25.2.10273 28439333 PMC5390067

[12] Yeoh PL, Hornetz K, Dahlui M. Antenatal care utilisation and content between low-risk and high-risk pregnant women. PLoS One. 2016;11(3):e0152167. doi: 10.1371/journal.pone.0152167 27010482 PMC4807004

[13] Agha S, Tappis H. The timing of antenatal care initiation and the content of care in Sindh, Pakistan. BMC pregnancy and childbirth. 2016;16:1–9. 27460042 10.1186/s12884-016-0979-8 PMC4962355

[14] Belayneh T, Adefris M, Andargie G. Previous early antenatal service utilization improves timely booking: cross-sectional study at university of Gondar hospital, northwest Ethiopia. Journal of pregnancy. 2014;2014. doi: 10.1155/2014/132494 25101176 PMC4102065

[15] Ziyo FY, Matly FA, Mehemd GM, Dofany EM. Relation between prenatal care and pregnancy outcome at Benghazi. Sudanese Journal of public health. 2009;4(4):403–10.

[16] Cantwell R, Clutton-Brock T, Cooper G, Dawson A, Drife J, Garrod D, et al. Saving Mothers’ Lives: Reviewing maternal deaths to make motherhood safer: 2006–2008. The Eighth Report of the Confidential Enquiries into Maternal Deaths in the United Kingdom. BJOG: an international journal of obstetrics and gynaecology. 2011;118:1–203. 10.1111/j.1471-0528.2010.02847.x 21356004

[17] Grum T, Brhane E. Magnitude and factors associated with late antenatal care booking on first visit among pregnant women in public health centers in central zone of Tigray Region, Ethiopia: A cross sectional study. PloS one. 2018;13(12):e0207922. doi: 10.1371/journal.pone.0207922 30517182 PMC6281255

[18] Oladokun A, Oladokun RE, Morhason-Bello I, Bello AF. Proximate predictors of early antenatal registration among Nigerian pregnant women. Annals of African Medicine. 2010;9(4). 20935421 10.4103/1596-3519.70959

[19] ICF EPHIEEa. Ethiopia Mini Demographic and Health Survey 2019: Key Indicators. Rockville, Maryland, USA: EPHI and ICF, 2019.

[20] Central Statistical Agency (CSA) [Ethiopia] and ICF. Ethiopia Demographic and Health Survey 2016. 2016.

[21] Tesfaye G, Loxton D, Chojenta C, Semahegn A, Smith R. Delayed initiation of antenatal care and associated factors in Ethiopia: a systematic review and meta-analysis. Reproductive health. 2017;14:1–17. 29141675 10.1186/s12978-017-0412-4 PMC5688656

[22] Teshale AB, Tesema GA. Prevalence and associated factors of delayed first antenatal care booking among reproductive age women in Ethiopia; a multilevel analysis of EDHS 2016 data. PloS one. 2020;15(7):e0235538. doi: 10.1371/journal.pone.0235538 32628700 PMC7337309

[23] Tessema GA, Laurence CO, Melaku YA, Misganaw A, Woldie SA, Hiruye A, et al. Trends and causes of maternal mortality in Ethiopia during 1990–2013: findings from the Global Burden of Diseases study 2013. BMC public health. 2017;17:1–8. 28152987 10.1186/s12889-017-4071-8 PMC5290608

[24] Ethiopian Public Health Institute (EPHI) E, Ministry of Health (MoH) [Ethiopia] and ICF. Ethiopia Service Provision Assessment 2021–22 Final Report. 2023.

[25] Gudayu TW, Woldeyohannes SM, Abdo AA. Timing and factors associated with first antenatal care booking among pregnant mothers in Gondar Town; North West Ethiopia. BMC pregnancy and childbirth. 2014;14(1):1–7. doi: 10.1186/1471-2393-14-287 25154737 PMC4152591

[26] Darmawan I, Keeves JP. Suppressor variables and multilevel mixture modelling. International Education Journal. 2006;7(2):160–73.

[27] Goldstein H. Multilevel statistical models: John Wiley & Sons; 2011.

[28] Moller A-B, Petzold M, Chou D, Say L. Early antenatal care visit: a systematic analysis of regional and global levels and trends of coverage from 1990 to 2013. The Lancet Global Health. 2017;5(10):e977–e83. doi: 10.1016/S2214-109X(17)30325-X 28911763 PMC5603717

[29] Gudayu TW. Proportion and factors associated with late antenatal care booking among pregnant mothers in Gondar town, north West Ethiopia. African journal of reproductive health. 2015;19(2):93–9. 26506661

[30] Gebresilassie B, Belete T, Tilahun W, Berhane B, Gebresilassie S. Timing of first antenatal care attendance and associated factors among pregnant women in public health institutions of Axum town, Tigray, Ethiopia, 2017: a mixed design study. BMC pregnancy and childbirth. 2019;19:1–11. 31533657 10.1186/s12884-019-2490-5 PMC6751589

[31] Aliyu AA, Dahiru T. Predictors of delayed Antenatal Care (ANC) visits in Nigeria: secondary analysis of 2013 Nigeria Demographic and Health Survey (NDHS). The Pan African medical journal. 2017;26:124. Epub 2017/05/24. doi: 10.11604/pamj.2017.26.124.9861 . 28533847 PMC5429423

[32] Sinyange N, Sitali L, Jacobs C, Musonda P, Michelo C. Factors associated with late antenatal care booking: population based observations from the 2007 Zambia demographic and health survey. The Pan African Medical Journal. 2016;25. doi: 10.11604/pamj.2016.25.109.6873 28292072 PMC5325499

[33] Manyeh AK, Amu A, Williams J, Gyapong M. Factors associated with the timing of antenatal clinic attendance among first-time mothers in rural southern Ghana. BMC pregnancy and childbirth. 2020;20(1):1–7. doi: 10.1186/s12884-020-2738-0 31959137 PMC6972022

[34] Kondale M, Tumebo T, Gultie T, Megersa T, Yirga H. Timing of first antenatal care visit and associated factors among pregnant women attending anatal clinics in Halaba Kulito governmental health institutions, 2015. J Women’s Health Care. 2016;5(308):2167–0420.

[35] Gulema H, Berhane Y. Timing of first antenatal care visit and its associated factors among pregnant women attending public health facilities in Addis Ababa, Ethiopia. Ethiopian journal of health sciences. 2017;27(2):139–46. doi: 10.4314/ejhs.v27i2.6 28579709 PMC5440828

[36] Aung TZ, Oo WM, Khaing W, Lwin N, Dar HT. Late initiation of antenatal care and its determinants: a hospital based cross-sectional study. Int J Community Med Public Health. 2016;3(4):900–5.

[37] Tsegaye B, Ayalew M. Prevalence and factors associated with antenatal care utilization in Ethiopia: an evidence from demographic health survey 2016. BMC Pregnancy and Childbirth. 2020;20(1):1–9. doi: 10.1186/s12884-020-03236-9 32917156 PMC7488553

[38] Tekelab T, Chojenta C, Smith R, Loxton D. Factors affecting utilization of antenatal care in Ethiopia: a systematic review and meta-analysis. PloS one. 2019;14(4):e0214848. doi: 10.1371/journal.pone.0214848 30973889 PMC6459485

[39] Gidey G, Hailu B, Nigus K, Hailu T, G/her W, Gerensea H. Timing of first focused antenatal care booking and associated factors among pregnant mothers who attend antenatal care in Central Zone, Tigray, Ethiopia. BMC research notes. 2017;10:1–6. 29162155 10.1186/s13104-017-2938-5 PMC5699019

[40] Muyunda B, Makasa M, Jacobs C, Musonda P, Michelo C. Higher educational attainment associated with optimal antenatal care visits among childbearing women in Zambia. Frontiers in public health. 2016;4:127. doi: 10.3389/fpubh.2016.00127 27379228 PMC4909780

[41] Tripathy A, Mishra PS. Inequality in time to first antenatal care visits and its predictors among pregnant women in India: an evidence from national family health survey. Scientific Reports. 2023;13(1):4706. doi: 10.1038/s41598-023-31902-3 36949163 PMC10033916

[42] Bhatia M, Dwivedi L, Banerjee K, Bansal A, Ranjan M, Dixit P. Pro-poor policies and improvements in maternal health outcomes in India. BMC Pregnancy and Childbirth. 2021;21(1):389. doi: 10.1186/s12884-021-03839-w 34011316 PMC8135986

[43] Gong E, Dula J, Alberto C, de Albuquerque A, Steenland M, Fernandes Q, et al. Client experiences with antenatal care waiting times in southern Mozambique. BMC health services research. 2019;19(1):1–9. 31370854 10.1186/s12913-019-4369-6 PMC6670125

[44] Alem AZ, Yeshaw Y, Liyew AM, Tesema GA, Alamneh TS, Worku MG, et al. Timely initiation of antenatal care and its associated factors among pregnant women in sub-Saharan Africa: A multicountry analysis of Demographic and Health Surveys. PloS one. 2022;17(1):e0262411. doi: 10.1371/journal.pone.0262411 35007296 PMC8746770

[45] Fekadu GA, Ambaw F, Kidanie SA. Facility delivery and postnatal care services use among mothers who attended four or more antenatal care visits in Ethiopia: further analysis of the 2016 demographic and health survey. BMC pregnancy and childbirth. 2019;19:1–9. 30744583 10.1186/s12884-019-2216-8 PMC6371418

[46] Pervin J, Moran A, Rahman M, Razzaque A, Sibley L, Streatfield PK, et al. Association of antenatal care with facility delivery and perinatal survival—a population-based study in Bangladesh. BMC pregnancy and childbirth. 2012;12:1–12. 23066832 10.1186/1471-2393-12-111 PMC3495045

[47] Boah M, Mahama AB, Ayamga EA. They receive antenatal care in health facilities, yet do not deliver there: predictors of health facility delivery by women in rural Ghana. BMC pregnancy and childbirth. 2018;18(1):1–10. 29724178 10.1186/s12884-018-1749-6 PMC5934813

[48] Hernández-Vásquez A, Chacón-Torrico H, Bendezu-Quispe G. Prevalence of home birth among 880,345 women in 67 low-and middle-income countries: a meta-analysis of demographic and health surveys. SSM-population Health. 2021;16:100955. doi: 10.1016/j.ssmph.2021.100955 34805477 PMC8581368

[49] Chukwuma A, Wosu AC, Mbachu C, Weze K. Quality of antenatal care predicts retention in skilled birth attendance: a multilevel analysis of 28 African countries. BMC pregnancy and childbirth. 2017;17(1):1–10. 28545422 10.1186/s12884-017-1337-1 PMC5445515

[50] Jiwani SS, Amouzou-Aguirre A, Carvajal L, Chou D, Keita Y, Moran AC, et al. Timing and number of antenatal care contacts in low and middle-income countries: analysis in the countdown to 2030 priority countries. Journal of global health. 2020;10(1). doi: 10.7189/jogh.10.010502 32257157 PMC7101027

[51] Muluneh AG, Kassa GM, Alemayehu GA, Merid MW. High dropout rate from maternity continuum of care after antenatal care booking and its associated factors among reproductive age women in Ethiopia, Evidence from Demographic and Health Survey 2016. PloS one. 2020;15(6):e0234741. 32530948 10.1371/journal.pone.0234741 PMC7292400

[52] Ryan BL, Krishnan RJ, Terry A, Thind A. Do four or more antenatal care visits increase skilled birth attendant use and institutional delivery in Bangladesh? A propensity-score matched analysis. BMC Public Health. 2019;19:1–6. 31096959 10.1186/s12889-019-6945-4 PMC6521440

[53] Defar A, Getachew T, Taye G, Tadele T, Getnet M, Shumet T, et al. Quality antenatal care services delivery at health facilities of Ethiopia, assessment of the structure/input of care setting. BMC health services research. 2020;20:1–9. doi: 10.1186/s12913-020-05372-6 32487097 PMC7268345

[54] Balehegn M, Balehey S, Fu C, Liang W. Indigenous weather and climate forecasting knowledge among Afar pastoralists of north eastern Ethiopia: Role in adaptation to weather and climate variability. Pastoralism. 2019;9(1):1–14.

[55] Biza N, Mohammed H. Pastoralism and antenatal care service utilization in Dubti District, Afar, Ethiopia, 2015: a cross-sectional study. Pastoralism. 2016;6:1–7.

[56] Moorhead SA, Hazlett DE, Harrison L, Carroll JK, Irwin A, Hoving C. A new dimension of health care: systematic review of the uses, benefits, and limitations of social media for health communication. Journal of medical Internet research. 2013;15(4):e1933. doi: 10.2196/jmir.1933 23615206 PMC3636326

[57] Sicsic J, Franc C. Obstacles to the uptake of breast, cervical, and colorectal cancer screenings: what remains to be achieved by French national programmes? BMC health services research. 2014;14(1):1–12. 25282370 10.1186/1472-6963-14-465 PMC4282512

